# Land-Use Conversion Changes the Multifractal Features of Particle-Size Distribution on the Loess Plateau of China

**DOI:** 10.3390/ijerph13080785

**Published:** 2016-08-05

**Authors:** Caili Sun, Guobin Liu, Sha Xue

**Affiliations:** 1 State Key Laboratory of Soil Erosion and Dryland Farming on the Loess Plateau of Northwest A & F University, Yangling 712100, Shaanxi, China; suncaili2007@126.com (C.S.); gbliu@ms.iswc.ac.cn (G.L.); 2 Institute of Soil and Water Conservation, Chinese Academy of Sciences and Ministry of Education, Yangling 712100, Shaanxi, China

**Keywords:** particle-size distribution, Grain for Green project, multifractal feature, soil erosion

## Abstract

Analyzing the dynamics of soil particle-size distributions (PSDs), soil nutrients, and erodibility are very important for understanding the changes of soil structure and quality after long-term land-use conversion. We applied multifractal Rényi spectra (*D*_q_) and singularity spectra (*f*(α)) to characterize PSDs 35 years after conversions from cropland to shrubland with *Caragana microphylla* (shrubland I), shrubland with *Hippophae rhamnoides* (shrubland II), forested land, and grassland on the Loess Plateau of China. Multifractal parameters (capacity dimension (*D*_0_), entropy dimension (*D*_1_), *D*_1_/*D*_0_, correlation dimension (*D*_2_), and Hölder exponent of order zero (α_0_)) were used to analyze the changes of PSDs. *D_q_* and *f*(α) characterized the PSDs well and sensitively represented the changes in PSDs after conversion. All types of land-use conversion significantly improved the properties of the topsoil (0–10 cm), but the effect of shrubland I and even forested land decreased with depth. All types of land-use conversion significantly increased *D*_1_ and *D*_2_ in the topsoil, and *D*_1_ and *D*_2_ in the 10–50 cm layers of shrubland II, forested land, and grassland and *D*_1_ in the 50–100 cm layers of shrubland II were significantly higher relative to the control. Both *D*_1_ and *D*_2_ were positively correlated with the contents of soil nutrients and fine particles and were negatively correlated with soil erosion, indicating that *D*_1_ and *D*_2_ were potential indices for quantifying changes in soil properties and erosion. In conclusion, all types of land-use conversion significantly improved the conditions of the topsoil, but conversion from cropland to shrubland II, forested land, and grassland, especially shrubland II and grassland, were more effective for improving soil conditions in deeper layers.

## 1. Introduction

The Loess Plateau of China covers approximately 62.4 × 10^4^ km^2^ [1], and is known for its long agricultural history and serious soil erosion [1,2]. The erosion has led to massive losses of soil and water, soil degradation, and ecological deterioration [3,4]. The Chinese government launched the state-funded “Grain for Green” project in 1999, which encouraged and facilitated the conversion of croplands with slopes >15° to forests and grasslands to control soil erosion and restore the ecological environment [2,3,4]. Forested land, shrubland, and grassland are now common on the Loess Plateau.

Soil erodibility is an important factor determining the rate of soil loss and is represented by an erodibility factor (*K*). *K* can be directly measured by the long-term monitoring of erosion in runoff plots, which is accurate but costly and time-consuming [5,6]. Techniques have been developed to estimate *K* from readily available data for soil properties. *K* estimated with the Erosion-Productivity Impact Calculator (EPIC) model is of particular interest to this study and was calculated using soil organic-carbon content and particle-size distribution (PSD) [7]. Zhang et al. (2008) reported that measured *K* and the *K* estimated from data for soil properties in China were strongly linearly correlated, although the calculated values were systematically higher than the measured values [8]. Recent extensive studies have evaluated the effectiveness of land-use conversion and have demonstrated that conversion from sloped cropland to green land greatly contributed to the improvement of soil conditions and effectively protected against erosion [9,10,11]. These positive effects of conversion may also have a large influence on the distribution of soil particles, because changes in soil conditions and erodibility can intimately affect fractions of fine particles [4,12].

PSD is a fundamental physical attribute of soil that greatly influences soil-moisture migration, solution transformation and soil erosion [13,14,15]. Characterizing its variation is therefore important for understanding and quantifying soil structure and dynamics. Multifractal analysis using laser-diffraction technology is commonly used by pedologists worldwide to characterize PSDs [16,17,18]. Multifractal analysis provides detailed information about the heterogeneity of a PSD [12,19], and this information can be expressed using multifractal spectra and parameters. The Rényi spectrum (*D_q_*) and singularity spectrum (*f*(α)) are used to characterize the scaling properties [20] and heterogeneity of PSDs [21], respectively. Multifractal parameters mainly include the capacity dimension (*D*_0_), entropy dimension (*D*_1_), correlation dimension (*D*_2_) and Hölder exponent of order zero (α_0_), which synthetically characterize PSD systems [18,20,22] (see the Materials and Methods section for details). Many studies have successfully applied multifractal spectra and parameters for characterizing the changes of PSDs in soils of the same or different texture. Martín and Montero (2002) proposed that *f*(α) was useful for the characterization of PSDs [23]. Wang et al. (2008) reported that *D*_0_ and *D*_1_ were significantly influenced by land-use on the Loess Plateau [12]. Paz-Ferreiro et al. (2010) found that the multifractal analysis of PSDs could provide further insight for verifying variation between plots [17]. Multifractal analysis is therefore a potential tool of great interest for detecting modifications of soil physical properties due to changes in land management, such as type of cultivation and land-use conversion.

Many studies of the effect of land-use conversion on the Loess Plateau of China have focused mainly on the biochemical rather than the physical properties of soil [2,9,24]. Wang et al. (2008) studied the multifractal characteristics of PSD under different land-use types but mainly studied the effect of land-use and topographical factors on multifractal parameters [12]. The changes in the multifractal features and their causes have not been well studied, and few reports addressing these scientific questions are yet available, which was the motivation behind this study. We therefore analyzed soil-nutrient contents, erodibility, and the multifractal features of PSDs of five common land-use types on the Loess Plateau approximately 35 years after their conversion. The goals of this study were to: (1) assess the effect of land-use conversion on soil nutrients, erodibility, and PSDs using multifractal analysis; and (2) identify the causes of the changes in soil PSDs. 

## 2. Materials and Methods

### 2.1. Study Area

**Figure 1:**
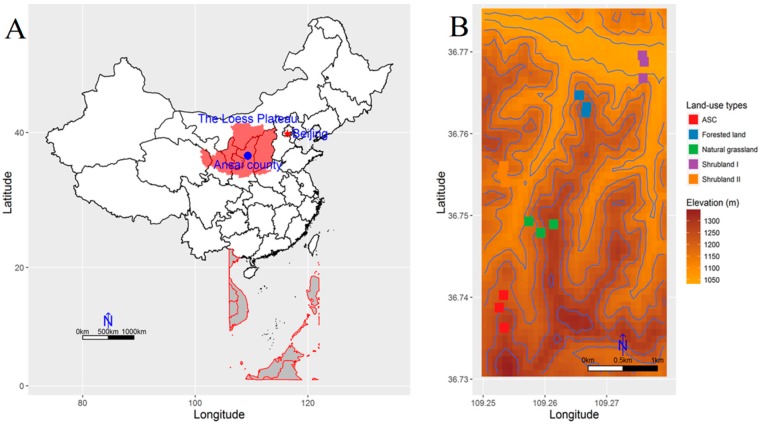
Study area and soil-sample sites. (**A**) Location of the Loess Plateau of China and Ansai county; (**B**) distribution of the soil-sample sites in the various land-use types.

This study was conducted in Ansai County (36°31′–37°20′ N and 108°52′–109°26′ E, 1010–1431 m a.s.l.) in Shaanxi Province in the central part of the Loess Plateau (Figure 1), which is well known for its high rates of erosion [25]. This area has a typical semiarid continental climate with a mean annual temperature of 8.8 °C and a mean annual precipitation of 549.1 mm, but temperature and precipitation are highly variable (74.3% of the rain falls between June and September). The landform is a typical hilly-gullied loessial landscape with deeply incised gullies. The soil is a Calcaric Regosol in the WRB reference system [26], originating from wind-blown deposits, and is characterized by a yellow color, absence of bedding, silty texture, looseness, macroporosity, and wetness-induced collapsibility [5]. These characteristics have contributed greatly to the severe soil erosion.

Substantial efforts have been made by the Chinese government since the 1950s to control soil erosion on the Loess Plateau, but the effects were not evident [2]. The state-funded “Grain for Green” project was initiated in 1999 to control soil erosion and restore the ecological environment. All croplands with slopes >15° were converted to green land [27]. The typical vegetation in the study area presently includes herbs such as *Stipa bungeana* and *Artemisia sacrorum*, shrubs such as *Caragana microphylla* and *Hippophae rhamnoides*, and woody plants such as *Robinia pseudoacacia* [25,27].

### 2.2. Experimental Design and Soil Sampling

**Figure 2:**
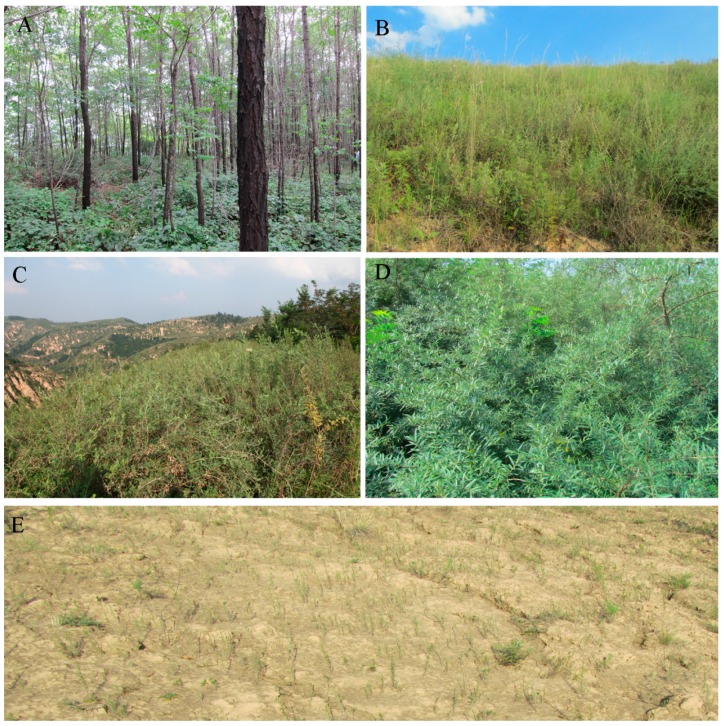
Photographs of the land-use types. (**A**) Forested land with *Robinia pseudoacacia*; (**B**) grassland with *Stipa bungeana* and *Artemisia sacrorum*; (**C**) shrubland I with *Caragana microphylla*; (**D**) shrubland II with *Hippophae rhamnoides*; and (**E**) sloped cropland abandoned for <1 year.

The five commonest land-use types in this region were selected in September 2011: forested land, shrubland with *Caragana korshinskii* (Shrubland I), shrubland with *Hippophae rhamnoides* (Shrubland II), grassland, and abandoned sloped cropland (ASC) that had been abandoned for less than one year for comparison (Figure 2). We selected the ASC location rather than an active sloped cropland to reduce the influence of human disturbance on soil PSDs. The selected locations had been subjected to similar farming practices before the initiation of the ecological project and had been revegetated approximately 35 years ago. The forested land was revegetated mainly with *Robinia pseudoacacia*, and the grassland was mainly covered by perennial *Stipa bungeana* and *Artemisia sacrorum*. Each land-use is described in Table 1.

Soil samples were collected from a total of 15 sites, three for each land-use type (Figure 1B). These sites had similar topographic characteristics (elevations and slopes), and all investigated soils had developed from the same parental materials. Soil samples were collected at each site from four randomly selected points using a soil auger (4 cm diameter). The samples were separately collected from five layers, 0–10, 10–20, 20–30, 30–50, and 50–100 cm, and the samples from the four points for the same layer were mixed to produce a composite sample for each layer and site. The mixed samples were air-dried and passed through both a 0.25 and a 1.0 mm sieve after removing the roots, stones, and debris for the determination of soil organic carbon (SOC), total nitrogen (TN), and total phosphorus (TP) contents and PSDs.

### 2.3. Laboratory Analysis

SOC was determined by the Walkley–Black method [28], TN by the Kjeldahl method [29], and TP by molybdenum antimony blue colorimetry [30]. Soil PSDs were analyzed by laser diffraction using a Longbench Mastersizer 2000 (Malvern Instruments, Malvern, UK). PSD was described based on the percentages of clay (<0.002 mm), fine silt (0.002–0.02 mm), coarse silt (0.02–0.05 mm), and sand (0.05–1 mm). Clay and fine silt were considered fine soil particles and coarse silt and sand were considered coarse particles. 

### 2.4. Multifractal Theory 

Multifractal analysis of particle distributions over an interval of sizes *I* commonly uses successive partitions of the interval in dyadic scaling down [31]. With a diameter *L* of interval *I*, dyadic partitions in *k* stages (*k* = 1, 2, 3, …) generate a number of cells *N*(ε) = 2*^k^* with diameter ε = *L* × *2^−k^* that cover the initial interval *I*. At every size scale ε, a number *N*(ε) = 2*^k^* of cells are considered, and their respective measures, μ*_i_*(ε), are supplied by the available data. For PSDs, μ*_i_*(ε) in each subinterval of sizes represented the relative volume of soil particles of characteristic size in the subinterval [20,32].

This study considered the array of particle sizes of *I* = [0.12, 724.44 μm], which was subdivided into 64 subintervals *I_i_* = [*ϕ_i_*, *ϕ_i+1_*], *i* = 1, 2, 3, …, 64. The length of the subintervals follows a logarithmic scale, and log(*ϕ_i_*_+1_/*ϕ_i_*) is constant, i.e., the first subinterval is *I*_1_ = [0.12, 0.137], and the last subinterval is *I*_64_ = [632.30, 724.44]. For constructing a new measure where multifractal techniques can be applied to take advantage of the data potential, a transformation such as φ_j_ = log(*ϕ_j_*/*ϕ*_1_), for *j* = 1, 2,…, 65, can create a new dimensionless interval *J* = [0, 3.78] partitioned into 64 subintervals of equal length [12,23]. ε then has a value of *J* × 2^−*k*^ for *k* ranging from 1 to 6, that is ε = 1.89 to 0.06. 

The Rényi dimension, *D_q_*, can be calculated by equations proposed by Hentschel and Procaccia (1983) [33]:

(1) $$D_{q}=\frac{1}{q-1}\underset{\varepsilon \to 0}{lim}\frac{log\;[\sum_{i\;=\;1}^{\;N\left(\varepsilon \right)}\mu_{i}^{q}\left(\varepsilon \right)]}{log\varepsilon }\;\left(q\neq 1\right)\;$$

and

(2) $$D_{1}=\underset{\varepsilon \to 0}{lim}\frac{\sum_{i\;=\;1}^{\text{N}\;\left(\varepsilon \right)}\mu_{i}\left(\varepsilon \right)log\mu_{i}\left(\varepsilon \right)}{log\varepsilon }\;\left(q=1\right)$$

The most frequently used generalized dimensions are *D*_0_ for *q* = 0, *D*_1_ for *q* = 1, and *D*_2_ for *q* = 2, which are termed the capacity, entropy, and correlation dimensions, respectively [18,20]. *D*_0_ provides general information of the PSD system; when *D*_0_ = 1, all intervals or cells would have some abundance of particle volume under successively finer partitions, whereas all subintervals are empty when *D*_0_ = 0 [18]. *D*_1_ is directly associated with the entropy of the system and is also a measure of the heterogeneity of a PSD. A higher *D*_1_ indicates higher heterogeneity of the PSD [12,20]. *D*_1_/*D*_0_ has been suggested to indicate the heterogeneity of PSDs; a value near 1 indicates sets with similar dimensions, and a value near 0 indicates that most of the measure is concentrated in a small region of the set of sizes [11,19]. *D*_2_ describes the uniformity of the measured values among intervals [22]. 

Following Chhabra and Jensen (1989) [34], the singularity spectrum can be calculated by a set of real numbers, *q*, by:

(3) $$\alpha \left(q\right)=\underset{\varepsilon \to 0}{lim}\frac{\sum_{i\;=\;1}^{N\left(\varepsilon \right)}\mu_{i}\left(q,\;\varepsilon \right)log\mu_{i}\left(\varepsilon \right)}{log\varepsilon }$$

and

(4) $$f\left(\alpha \right)=\underset{\varepsilon \to 0}{lim}\frac{\sum_{i\;=\;1}^{N\left(\varepsilon \right)}\mu_{i}\left(q,\varepsilon \right)log\;\mu_{i}\left(q,\;\varepsilon \right)}{log\varepsilon }$$

where $\mu_{i}\left(q,\;\varepsilon \right)=\mu_{i}{\left(\varepsilon \right)}^{q}\;/\;\sum_{i\;=\;1}^{N\left(\varepsilon \right)}\mu_{i}{\left(\varepsilon \right)}^{q}$ [31].

The value of α_0_ quantifies the average scale of local mass density, i.e., α_0_ is the average of the singularity strength of the PSDs [21]. A high value of α_0_ corresponds to PSDs exhibiting, on average, a low degree of volume concentration, and the opposite is also true. 

The singularity spectrum, *f*(α), provides the entropy dimension of the distorted measure μ(*q*, ε) and characterizes the original measure μ by analyzing the variation under successive distortions driven by *q* [23]. *f*(α) is maximum when *q* = 0 and typically has a parabolic shape around this point. *q* provides a tool for inspecting the denser and rarer regions of μ. When *q* >> 1, regions where μ is highly concentrated are amplified, and *q* << −1 indicates the amplification of regions where μ is less concentrated. Finally, the measure itself is replicated when *q* = 1. 

### 2.5. Erodibility (K)

*K* is calculated using SOC content and soil PSD in the EPIC model [7] as:

(5) $$K\;=\;\left\{0.2+0.3\;exp[-0.0256SAN\left(1-0.01SIL\right)]\right\}\times {\left[SIL/\left(CLA+SIL\right)\right]}^{0.3}\times \;\;\left\{1.0-0.25C/\left[C+\text{exp}\left(3.72-2.95C\right)\right]\right\}\times \left\{1.0-0.7\text{SNI}/\left[\text{SNI}+\text{exp}\left(-5.51+22.9\text{SNI}\right)\right]\right\}$$

where SAN, SIL, and CLA are the sand (%), silt (%), and clay (%) fractions, respectively. *C* is the SOC content (%), and SNI = 1 − SAN/100.

### 2.6. Statistical Analysis

The data in the figures and tables are means for each sample. All statistical analyses were implemented using various packages within the R statistical computing environment (R version 3.2.3). One-way analyses of variation compared the effects of land-use type and soil depth on the soil nutrients, texture, and multifractal parameters. Duncan’s tests separated the means of these variables at *p* < 0.05. 

Network analyses were used to show the composition of, and interactions between, multiple elements in the communities. A matrix of the correlations between all trait pairs was generated by network analysis based on significance levels at *p* < 0.05 or on both the significant level and correlation coefficients of *r* > 0.4. These pairs were considered as a network in which a vertex corresponds to a trait, and a link between two vertices corresponds to significant correlations between these two traits. This network was then plotted based on the adjacency matrix using the R *igraph* package. 

## 3. Results

### 3.1. Effects of Land-Use Type and Soil Depth on Soil Nutrient Content and Texture

SOC, TN, clay, and fine-silt contents in the 0–10 cm layer (topsoil) were significantly higher and the coarse-silt content and erodibility were significantly lower in all older land-use types compared to ASC. In the 10–30 cm layers (shallow soil), SOC and TN contents in shrubland II and grassland, clay content in all older land-use types, and fine-silt content in shrubland II, forested land, and grassland were significantly higher, whereas coarse-silt content and erodibility in all older land-use types were significantly lower, relative to ASC. In the 30–100 cm layers (deep soil), SOC, clay, and fine-silt contents in shrubland II and grassland were significantly higher and sand content in grassland was significantly lower than in ASC (Table 2).

SOC and TN contents generally tended to decrease with soil depth and remained unchanged below 30 cm. TP content was highest in shrubland I and forested land. Changes of soil texture with soil depth were not obvious but were observed in several of the land-use types. 

### 3.2. Effects of Land-Use Type and Soil Depth on Soil Erodibility

**Figure 3:**
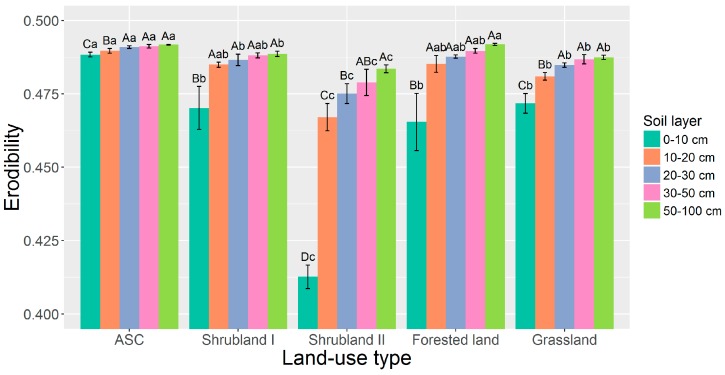
Effect of land-use conversion and soil depth on erodibility. Numerical values are means ± standard deviation for three samples. Different uppercase letters indicate significant differences between depths within each treatment, and different lowercase letters indicate significant differences between treatments at each depth (Duncan’s test, *p* < 0.05). ASC: sloped cropland abandoned for <1 year.

The erodibility in the 0–10 cm layer of all older land-use types and in the 10–100 cm layers of shrubland II and grassland were significantly lower than in ASC (Figure 3). Erodibility generally tended to increase with soil depth and remained unchanged below 20 cm. 

### 3.3. Multifractal Characterization of Soil PSDs

**Figure 4:**
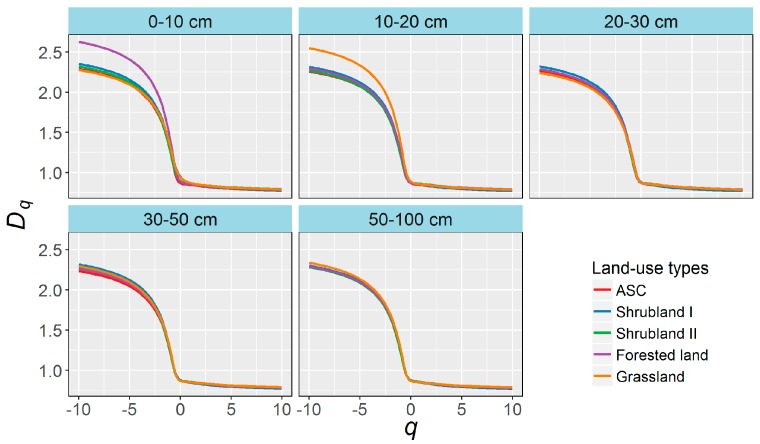
Rényi spectra (*D_q_*) of randomly selected samples in each soil layer of the various land-uses.

Selected examples of Rényi spectra (*D*_q_) are shown in Figure 4. These curves were calculated for 0.2 *q* steps in the range of moments from −10 to 10. The *D_q_* spectra were typical sigmoidal curves, notably decreased as *q* increased, and differed in shape among land-use types in both the 0–10 and 10–20 cm layers.

**Figure 5:**
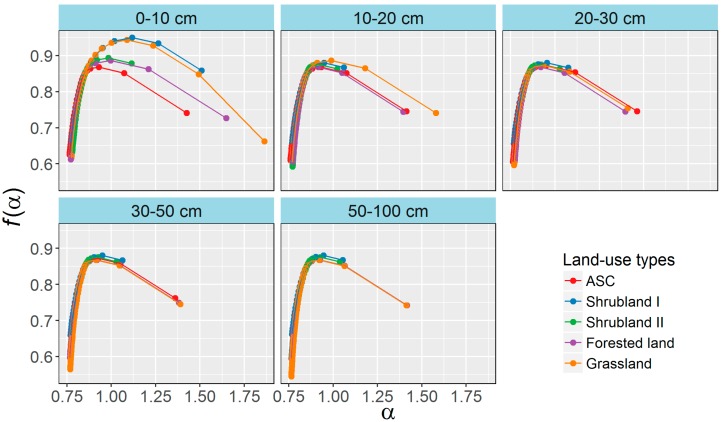
Singularity spectra (*f*(α)) of randomly selected samples in each soil layer of the various land-uses.

Singularity spectra, *f*(α), of selected samples are shown in Figure 5. The shapes of the *f*(α) spectra of the selected samples varied greatly among the land-use types for the same soil layers, especially the 0–10 and 10–20 cm layers, even though the spectra were characterized by a typical concave downward shape. All *f*(α) spectra had long left components, but the right components were quite different.

### 3.4. Effects of Land-Use Type and Soil Depth on the Multifractal Parameters

*D*_0_ in the 0–20 cm layers were significantly higher for shrubland I and grassland than the other land-use types. *D*_0_ did not differ significantly among the land-use types in the 20–100 cm layers. *D*_1_ in the 0–20 cm layers in all the older land-use types (i.e., not ASC); in the 20–50 cm layers of shrubland II, forested land, and grassland; and in the 50–100 cm layer of shrubland II were significantly higher than in ASC. *D*_1_/*D*_0_ differed significantly among the land-use types only in the 0–10 cm layers, where *D*_1_/*D*_0_ was lower in shrubland I and grassland than ASC. *D*_2_ was significantly higher in the 0–10 cm layers of all the older land-use types and in the 10–50 cm layers of shrubland II, forested land, and grassland than ASC. α_0_ was significantly higher in the 0–10 cm layers of shrubland I and grassland than ASC (Table 3). 

In shrubland I and grassland, *D*_0_, *D*_1_, *D*_2_, and α_0_ were significantly higher and *D*_1_/*D*_0_ was significantly lower in the 0–10 cm layer than the other layers. The multifractal parameters in the other three land-use types differed by minor significance with increasing depth (Table 3).

### 3.5. Relationships between the Multifractal Parameters and Selected Soil Properties and Erodibility

**Figure 6:**
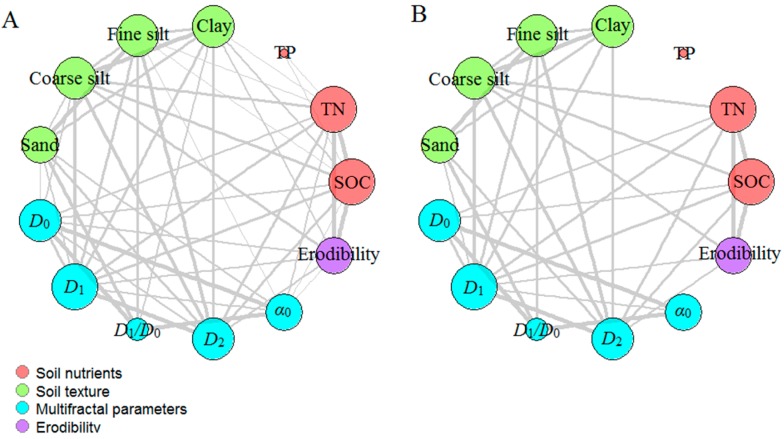
Network plot showing the associations between the multifractal parameters and selected soil properties and erodibility. (**A**) Significant correlations (*p* < 0.05); and (**B**) moderately or highly significant correlations (*r* > 0.4, *p* < 0.05).

**Figure 7:**
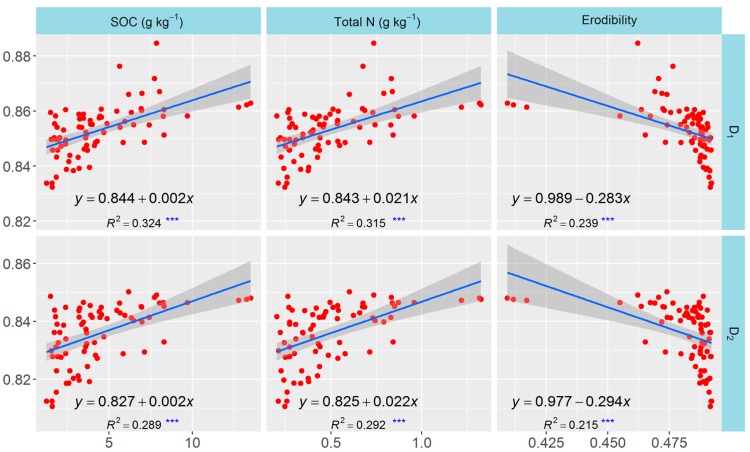
Relationships between multifractal parameters and SOC and TN contents and erodibility. *** Significant at *p* < 0.001.

**Figure 8:**
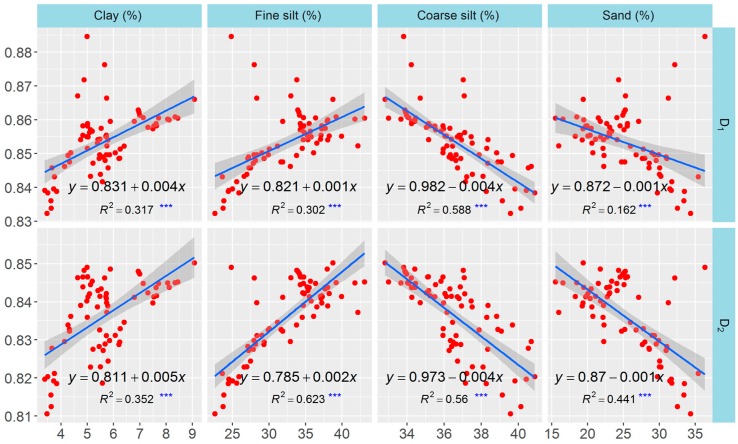
Relationships between multifractal parameters and soil texture. *** Significant at *p* < 0.001.

The correlation network including the traits of soil nutrients, texture, erodibility, and multifractal parameters were plotted to test the relationships among these parameters. All significant correlations (*p* < 0.05) were visualized as edges in the network (Figure 6A), and the network was simplified based on the correlation coefficients and statistical significance (Figure 6B, *r* > 0.4 and *p* < 0.05). The size of a vertex in the network was dependent on the node degree: the larger the node degree of one vertex, the stronger the connection with other vertices, indicating a more important corresponding trait in the network. The vertices for clay, fine-silt, coarse-silt, sand, SOC, and TN contents and for *D*_0_, *D*_1_, *D*_2_, and erodibility were obviously larger than those for the other parameters. Considering the importance of multifractal parameters and their relationships with other soil properties, *D*_1_ and *D*_2_ were selected for the analyses of linear regression to quantify the relationships. *D*_1_ and *D*_2_ were linearly correlated positively with SOC and TN contents but negatively with erodibility (*p* < 0.001) (Figure 7). *D*_1_ and *D*_2_ were correlated positively with clay and fine-silt contents, but correlated negatively with coarse-silt and sand contents (*p* < 0.001) (Figure 8). The slope coefficients of the regression lines were highest for the models between erodibility and *D*_1_ and *D*_2_ (−0.283 and −0.294, respectively), and the slope coefficients of the regression lines were higher for the models between clay content and *D*_1_ and *D*_2_ (0.004 and 0.005, respectively) than for the other particle-size classes.

## 4. Discussion

### 4.1. Land-Use Conversion Affects Soil Nutrients, Texture, and Erodibility

Land-use conversion from cropland to other land-use types can improve soil properties and decrease soil erosion, but with different effects. This finding was consistent with previous studies showing that conversion from sloped cropland to perennial vegetation could greatly improve soil nutrients [11] and SOC sequestration [25,35] and decrease the erosion of topsoil [9,36]. Our results also supported these observations, showing that all older land-use types had significantly higher SOC, TN, and fine-soil contents and lower topsoil erodibility. Soil-nutrient contents did not improve in shrubland I and forested land in the shallow soil layers, and SOC and fine-particle contents increased significantly while erodibility decreased significantly in shrubland II and grassland in deep soil layers. These changes were mainly due to the high degree of vegetation coverage and the high input of residues [2,24,37]. Stable ecosystems and new microclimatic regimes can be established after conversion in about 35 years [38], with well-developed root systems, dense canopies for forests and shrubland, and better vegetation coverage for grassland, with high amounts of ground litter. These traits can potentially improve soil conditions, decrease erosion of the topsoil by reducing raindrop splash and impeding surface runoff, which can consequently protect fine soil particles [13,39]. Soil nutrients did not significantly increase in shallow layers in shrubland I and forested land, likely because soil organic matter in these two land-use types is more easily oxidized or degraded by microorganisms under the specific microclimates. Sun et al. (2016) found relatively higher amounts of labile carbon in shrubland with *Caragana microphylla* [25]. SOC and fine-particle contents were significantly higher in the deep soil layers in shrubland II and grassland, indicating that these two land-use types facilitated the sequestration of soil carbon and improved the structure of deep soil. 

SOC and TN contents decreased significantly and erodibility increased significantly with depth, but changes of soil texture were not obvious. Previous studies in the same region reported similar results, showing that SOC and TN contents were significantly higher in the surface soil than in deeper layers [1,25,27]. The higher SOC and TN contents and lower erodibility were mainly attributed to the improvement of soil quality and structure. Residues such as litter and roots were more abundant and humic processes were more efficient in surface soil relative to deep soil, which facilitated the increase of soil nutrients and amelioration of soil physical properties. In contrast, relatively few residues are imported to deep soil, where many nutrients are absorbed by root systems to meet the needs for photosynthesis [2,37]. PSD is an essential physical attribute of a soil and is more stable compared to chemical properties, and textural classes can exhibit a wide range of PSDs [12,17], so associating changes of soil texture with soil depth is difficult. 

### 4.2. Land-Use Conversion Affects the Multifractal Features of Soil PSDs

The Rényi spectra (*D_q_*) and singularity spectra (*f*(α)) were able to characterize the PSDs well and sensitively identified the differences in PSDs among the land-use types. The *D_q_* spectra were typical sigmoidal curves and notably decreased as *q* increased, indicating that PSDs in the various land-use types presented multifractal rather than monofractal behavior. The different curves among the land-use types in the 0–10 and 10–20 cm layers suggested diverse scaling properties of these PSDs. The different shapes of the *f*(α) spectra suggested different heterogeneities of soil PSDs among the land-use types for the same soil layers, especially the 0–10 and 10–20 cm layers. The *D_q_* and *f*(α) spectra of the PSDs in the 0–20 cm layers changed significantly after 35 years of land-use conversion, indicating that land-use was an important factor in the distribution of soil particles, and these changes were determined well by the multifractal modes. The multifractal features of PSDs change mainly due to improvements of soil quality [2,25,37] and the reduction of erosion [9,36] of the surface soil, which generally facilitate the improvement of soil structure and increase the proportions of microaggregates and fine particles [40,41,42] and accordingly change the multifractal features of soil PSDs. 

Land-use conversion had a positive effect on the increase of the multifractal parameters such as *D*_0_, *D*_1_, *D*_2_, and α_0_, but *D*_1_/*D*_0_ changed little. *D*_0_ and α_0_ in shrubland I and grassland and *D*_1_ and *D*_2_ in all the older land-use types were significantly higher in the topsoil. *D*_1_ and *D*_2_ in the 10–50 cm layers of shrubland II, forested land, and grassland and *D*_1_ in the 50–100 cm layers of shrubland II were significantly higher than in ASC. As previously stated, different multifractal parameters represent different information for soil PSDs. High values of these multifractal parameters indicate that PSDs have wide ranges, high heterogeneities, and are more homogeneous among all intervals [21,43,44]. Increases in multifractal parameters may be attributed to increases in fine-soil particles [40,45,46]. The increase in fine-soil particles in the topsoil was likely due to the comprehensive effect of greatly improved soil quality and reduced soil erosion after the rehabilitation of the vegetation [10,38,47]. A high degree of vegetation coverage was attained 35 years after the land-use conversion, which could reduce soil erosion by reducing rain splash and could also improve the amount of soil organic matter and structure by increasing aboveground and root biomasses. High concentrations of soil organic matter and low soil erosion generally facilitate the improvement of soil structure and increase microaggregates and fine particles [5,13,39,48]. The penetration and fixation of developed root systems in shrublands, forested land, and grassland could also protect fine particles from erosion, thereby preserving the fine particles [4,13]. Increases in fine particles in non-topsoil layers, however, are mainly due to the improvement of soil quality and the interaction of root systems. Plant roots usually exude substrates, some of which are viscous, such as polysaccharides, phenolic compounds, and polygalacturonic acid that contribute greatly to the cohesion of fine soil particles [4]. In conclusion, land-use conversion facilitated the increase of fine particles and thus potentially contributed to the wide range of soil PSDs and the greater irregularity and heterogeneity of the PSDs. 

Soil depth had a significant effect on the multifractal parameters only in shrubland I and grassland. *D*_0_, *D*_1_, *D*_2_, and α_0_ were significantly higher, but *D*_1_/*D*_0_ was significantly lower, in the 0–10 cm than the other layers, suggesting that PSDs in the topsoil of these two land-use types were more heterogeneous relative to the deep soil layers. Higher multifractal parameters in the topsoil (0–10 cm) can be due to a higher content of fine particles [40]. The highest SOC content and lowest erodibility in the topsoil of these two land-use types may have greatly contributed to the higher content of fine particles relative to deep soil. Surface weathering can also contribute to an increase in fine particles [48]. The lower *D*_1_/*D*_0_ in the topsoil of shrubland I and grassland may have been largely due to the higher value of the denominator (*D*_0_). 

### 4.3. Multifractal Parameters Were Associated with the Selected Soil Properties and Erodibility

The network analysis clearly indicated that soil nutrients, texture, erodibility, and the multifractal parameters were significantly correlated with each other (*p* < 0.05) and that the node degrees for clay, fine-silt, coarse-silt, sand, SOC, and TN contents and *D*_1_, *D*_2_, and erodibility were larger than those for the other traits, indicating that these selected traits were more connected with the others and played more important roles in the network. The network could clearly identify the interrelationships but not the quantitative relationships between the multifractal parameters and the other soil properties. We eliminated *D*_0_, *D*_1_/*D*_0_, and α_0_ from the analyses of linear regression to simplify and better analyze these quantitative relationships. *D*_1_ and *D*_2_ were linearly and correlated positively with SOC, TN, clay, and fine-silt contents but negatively with erodibility and coarse-silt and sand contents (*p* < 0.001). This result suggested that *D*_1_ and *D*_2_ can be used as indicators of the changes in soil quality and erosion after land-use conversion, because SOC and TN contents are important indices for the assessment of soil quality and structural stability [48,49] and because erodibility calculated based on the EPIC model can estimate soil erosion well for the Loess Plateau [5,50]. Additionally, the effect of erodibility was largest on *D*_1_ and *D*_2_, and the effect of clay content on *D*_1_ and *D*_2_ was larger than for silt and sand contents, based on the slope coefficients of the models of linear regression. These results indicated that erodibility was the vital factor influencing multifractal characterization, so the effect of land-use conversion on the multifractal features of PSDs may mainly be due to a decrease in soil erosion, which would increase the clay content and the heterogeneity of the PDSs, thereby changing the multifractal features of the PSDs. 

## 5. Conclusions

The Rényi spectra (*D_q_*) and singularity spectra (*f*(α)) characterized the PSDs well and sensitively represented the changes of PSDs after land-use conversion. Land-use conversion greatly affected soil nutrients, erodibility, and the multifractal parameters. Specifically, all types of land-use conversion significantly improved the properties of the topsoil (0–10 cm), but the effect of shrubland I and even forested land decreased with depth. All types of land-use conversion significantly increased *D*_1_ and *D*_2_ in the topsoil, and *D*_1_ and *D*_2_ in the 10–50 cm layers of shrubland II, forested land, and grassland and *D*_1_ in the 50–100 cm layers of shrubland II were significantly higher relative to ASC. The other multifractal parameters also varied after land-use conversion but showed no clear trend. *D*_1_ and *D*_2_ were sensitive to the dynamics of soil nutrients and fine particles and could act as potential indices for quantifying changes in soil properties and erosion. Multifractal theory was therefore a useful tool for characterizing PSDs on the Loess Plateau of China. All types of land-use conversion significantly improved topsoil conditions, but conversion from cropland to shrubland II, forested land, and grassland, especially shrubland II and grassland, were more effective for improving the conditions in deeper soil layers.

## Figures and Tables

**Table 1:** Detailed information for the land-use types.

Land-Use Type	Restoration Age (Year)	Altitude (m)	Slope (°)	Main Vegetation or Crop Species
ASC	-	1270	19	-
Shrubland I	36	1211	20	*Caragana microphylla*
Shrubland II	35	1133	19	*Hippophae rhamnoides*
Forested land	37	1237	30	*Robinia pseudoacacia*
Grassland	35	1159	18	*Stipa bungeana*, *Artemisia sacrorum*

ASC: sloped cropland abandoned for <1 year.

**Table 2:** Effects of land-use type and soil depth on soil nutrients (g·kg^−1^) and soil texture (%).

Soil Property	Soil Layer (cm)	ASC	Shrubland I	Shrubland II	Forested Land	Grassland
SOC	0–10	3.652Ac	6.819 Ab	13.17 Aa	8.454 Ab	7.37 Ab
	10–20	3.263 ABc	3.871 Bc	7.844 Ba	4.477 Bbc	5.655 Bb
	20–30	2.493 BCc	3.084 BCbc	6.52 BCa	3.314 Bbc	4.173 BCb
	30–50	2.123 Cbc	2.186 Cbc	5.497 CDa	1.847 Cc	3.359 Cb
	50–100	1.744 Cc	1.793 Cc	4.234 Da	1.647 Cc	2.935 Cb
TN	0–10	0.468 Ad	0.734 Ac	1.287 Aa	0.886 Ab	0.8 Abc
	10–20	0.375 Bc	0.472 Bbc	0.788 Ba	0.552 Bb	0.626 Bb
	20–30	0.269 BCc	0.374 BCbc	0.646 BCa	0.457 Bb	0.453 Cb
	30–50	0.298 Cb	0.272 CDb	0.538 Ca	0.317 Cb	0.35 Cb
	50–100	0.249 Cab	0.227 Db	0.357 Da	0.276 Cab	0.299 Cab
TP	0–10	0.586	0.596 A	0.543	0.571 A	0.546
	10–20	0.579	0.546 B	0.601	0.559 AB	0.578
	20–30	0.564	0.545 B	0.569	0.537 AB	0.563
	30–50	0.553	0.532 B	0.574	0.522 B	0.562
	50–100	0.555	0.543 B	0.552	0.533 AB	0.539
Clay	0–10	4.133 c	5.46 b	6.965 a	4.956 Abc	5.431 ab
	10–20	3.898 c	5.819 b	7.678 a	5.441 Ab	5.731 b
	20–30	3.754 c	5.545 b	7.468 a	5.206 Ab	6.29 ab
	30–50	3.905 c	5.501 bc	7.602 a	5.366 Abc	6.14 ab
	50–100	3.886 c	5.478 b	7.065 a	3.886 Bc	6.386 ab
Fine silt	0–10	29.042 bc	27.061 c	34.054 a	34.876 a	32.89 Cab
	10–20	27.446 b	27.905 b	37.418 a	36.4 a	35.09 BCa
	20–30	27.367 b	27.409 b	36.368 a	35.201 a	36.765 ABa
	30–50	27.73 b	27.255 b	35.094 a	36.567 a	36.94 ABa
	50–100	29.022 b	28.14 b	34.318 ab	29.022 b	39.128 Aa
Coarse silt	0–10	38.396 a	34.241 Bcd	33.92 d	35.912 Bbc	36.117 b
	10–20	39.599 a	36.303 Ab	34.321 b	35.672 Bb	36.212 b
	20–30	39.75 a	36.849 Ab	34.853 c	35.69 Bbc	35.78 bc
	30–50	39.609 a	37.374 Aab	35.06 b	36.258 Bb	36.285 b
	50–100	39.837 a	37.431 Ab	35.815 b	39.837 Aa	36.105 b
Sand	0–10	28.429 ab	33.238 a	25.061 b	24.255 b	25.561 Ab
	10–20	29.058 a	29.974 a	20.583 b	22.487 ab	22.967 ABab
	20–30	29.13 a	30.197 a	21.312 b	23.903 ab	21.164 BCb
	30–50	28.755 ab	29.869 a	22.244 bc	21.81 bc	20.634 BCc
	50–100	27.256 a	28.952 a	22.801 ab	27.256 a	18.38 Cb

Numerical values are means ± standard deviation for three samples. Different uppercase letters within a column indicate significant differences between depths within each treatment, and different lowercase letters within a row indicate significant differences between treatments at each depth (Duncan’s test, *p* < 0.05). ASC: sloped cropland abandoned for <1 year.

**Table 3:** Effects of land-use type and soil depth on the multifractal parameters.

Parameter	Soil Layer (cm)	ASC	Shrubland I	Shrubland II	Forested Land	Grassland
*D*_0_	0–10	0.874 b	0.948 Aa	0.889 Ab	0.887 b	0.927 Aa
	10–20	0.871 b	0.885 Ba	0.877 Bab	0.871 b	0.884 Ba
	20–30	0.873	0.88 B	0.876 B	0.88	0.878 B
	30–50	0.871	0.88 B	0.877 B	0.876	0.874 B
	50–100	0.871	0.879 B	0.876 B	0.871	0.871 B
*D*_1_	0–10	0.846 c	0.876 Aa	0.862 b	0.857 Ab	0.866 Aab
	10–20	0.842 c	0.848 Bb	0.858 a	0.855 Aa	0.857 Ba
	20–30	0.842 b	0.847 Bb	0.856 a	0.857 Aa	0.857 Ba
	30–50	0.842 b	0.846 Bab	0.858 a	0.856 Aa	0.856 Ba
	50–100	0.843 b	0.848 Bab	0.856 a	0.843 Bb	0.855 Bab
*D*_1_/*D*_0_	0–10	0.968 a	0.924 Bb	0.970 a	0.967 a	0.935 Bb
	10–20	0.966 ab	0.959 Ab	0.978 a	0.982 a	0.970 Aab
	20–30	0.964 ab	0.962 Ab	0.978 a	0.973 ab	0.976 Aab
	30–50	0.966 ab	0.961 Ab	0.978 a	0.977 a	0.979 Aa
	50–100	0.968 ab	0.965 Ab	0.977 ab	0.968 ab	0.982 Aa
*D*_2_	0–10	0.829 b	0.845 Aa	0.848 a	0.845 Aa	0.845 a
	10–20	0.822 b	0.828 Bb	0.841 a	0.844 Aa	0.842 a
	20–30	0.823 b	0.827 Bb	0.839 a	0.844 Aa	0.843 a
	30–50	0.823 b	0.826 Bb	0.84 ab	0.844 Aa	0.841 ab
	50–100	0.825	0.828 B	0.839	0.825 B	0.839
α_0_	0–10	0.945 c	1.073 Aa	0.963 Ac	0.984 bc	1.059 Aab
	10–20	0.942	0.963 B	0.932 B	0.929	0.966 B
	20–30	0.95	0.955 B	0.928 B	0.959	0.949 B
	30–50	0.941	0.954 B	0.927 B	0.946	0.932 B
	50–100	0.941 ab	0.949 Ba	0.929 Bab	0.941 ab	0.92 Bb

Numerical values are means ± standard deviation for three samples. Different uppercase letters within a column indicate significant differences between depths within each treatment, and different lowercase letters within a row indicate significant differences between treatments at each depth (Duncan’s test, *p* < 0.05). ASC: sloped cropland abandoned for <1 year.

## References

[1] Fu X., Shao M., Wei X., Horton R. (2010). Soil organic carbon and total nitrogen as affected by vegetation types in Northern Loess Plateau of China. Geoderma.

[2] Chen L., Gong J., Fu B., Huang Z., Huang Y., Gui L. (2007). Effect of land-use conversion on soil organic carbon sequestration in the loess hilly area, Loess Plateau of China. Ecol. Res..

[3] Wang B., Liu G., Xue S., Zhu B. (2010). Changes in soil physico-chemical and microbiological properties during natural succession on abandoned farmland in the Loess Plateau. Environ. Earth Sci..

[4] Song Z., Zhang C., Liu G., Qu D., Xue S. (2015). Fractal feature of particle-size distribution in the rhizospheres and bulk soils during natural recovery on the Loess Plateau, China. PLoS ONE.

[5] Zhu B., Li Z., Li P., Liu G., Xue S. (2010). Soil erodibility, microbial biomass, and physical-chemical property changes during long-term natural vegetation restoration: A case study in the Loess Plateau, China. Ecol. Res..

[6] Zhang K., Li S., Peng W., Yu B. (2004). Erodibility of agricultural soils on the Loess Plateau of China. Soil Till. Res..

[7] Williams J.R., Jones C.A., Dyke P.T. (1984). A modeling approach to determining the relationship between erosion and productivity. Trans. ASAE.

[8] Zhang K., Shu A., Xu X., Yang Q., Yu B. (2008). Soil erodibility and its estimation for agricultural soils in China. J. Arid Environ..

[9] Sun W., Shao Q., Liu J., Zhai J. (2014). Assessing the effects of land-use and topography on soil erosion on the Loess Plateau in China. Catena.

[10] Zheng F. (2006). Effect of vegetation changes on soil erosion on the Loess Plateau. Pedosphere.

[11] Gong J., Chen L., Fu B., Huang Y., Huang Z., Peng H. (2006). Effect of land-use on soil nutrients in the loess hilly area of the Loess Plateau, China. Land Degrad. Dev..

[12] Wang D., Fu B., Zhao W., Hu H., Wang Y. (2008). Multifractal characteristics of soil particle size distribution under different land-use types on the Loess Plateau, China. Catena.

[13] Luo B., Chen X., Ding L., Huang Y., Zhou J., Yang T. (2015). Response characteristics of soil fractal features to different land-uses in typical purple soil watershed. PLoS ONE.

[14] Yu J.B., Lv X.F., Bin M., Wu H.F., Du S.Y., Zhou M., Yang Y.M., Han G.X. (2015). Fractal features of soil particle size distribution in newly formed wetlands in the Yellow River Delta. Sci. Rep.-UK.

[15] Chen X., Zhou J. (2013). Volume-based soil particle fractal relation with soil erodibility in a small watershed of purple soil. Environ. Earth Sci..

[16] Li Y., Li M., Horton R. (2011). Single and joint multifractal analysis of soil particle size distributions. Pedosphere.

[17] Paz-Ferreiro J., Vazquez E.V., Miranda J.G.V. (2010). Assessing soil particle-size distribution on experimental plots with similar texture under different management systems using multifractal parameters. Geoderma.

[18] Posadas A.N.D., Giménez D., Bittelli M., Vaz C.M.P., Flury M. (2001). Multifractal characterization of soil particle-size distributions. Soil Sci. Soc. Am. J..

[19] Gan X., Yang P., Ren S., Li X., Lv Y. (2009). Heterogeneity analysis of particle size distribution for loamy soil based on multifractal theory. J. Basic Sci. Eng..

[20] Montero E. (2005). Rényi dimensions analysis of soil particle-size distributions. Ecol. Model..

[21] Miranda J.G.V., Montero E., Alves M.C., Paz González A., Vidal Vázquez E. (2006). Multifractal characterization of saprolite particle-size distributions after topsoil removal. Geoderma.

[22] Paz-Ferreiro J., Marinho M.D., da Silva L.F.S., Motoshima S.T., Dias R.D. (2013). The Effects of bulk density and water potential on multifractal characteristics of soil penetration resistance microprofiles measured on disturbed soil samples. Vadose Zone J..

[23] Martín M.Á., Montero E. (2002). Laser diffraction and multifractal analysis for the characterization of dry soil volume-size distributions. Soil Tillage Res..

[24] Wang B., Xue S., Liu G., Zhang G., Li G., Ren Z. (2012). Changes in soil nutrient and enzyme activities under different vegetations in the loess Plateau area, Northwest China. Catena.

[25] Sun C., Xue S., Chai Z., Zhang C., Liu G. (2016). Effects of land-use types on the vertical distribution of fractions of oxidizable organic carbon on the Loess Plateau, China. J. Arid Land..

[26] IUSS Working Group WRB (2006). World Reference Base for Soil Resources 2006.

[27] Zhang C., Liu G., Xue S., Zhang C. (2012). Rhizosphere soil microbial properties on abandoned croplands in the loess Plateau, China during vegetation succession. Eur. J. Soil Biol..

[28] Nelson D.W., Sommers L.E., Page A.L., Miller R.H., Keeney D.R. (1982). Total carbon, organic carbon, and organic matter. Methods of Soil Analysis, Part 2.

[29] Bremner J.M., Mulvaney C.S., Page A.L., Miller R.H., Keeney D.R. (1982). Nitrogen-total. Methods of Soil Analysis, Part 2.

[30] Murphy J., Riley J.P. (1962). A modified single solution method for the determination of phosphate in natural waters. Anal. Chim. Acta.

[31] Evertsz C.J.G., Mandelbrot B.B., Peitgen H.O., Jürgens H., Saupe D. (1992). Multifractal measures, in Chaos and fractals. New Frontiers of Science.

[32] Martin M.A., Taguas F.J. (1998). Fractal modelling, characterization and simulation of particle-size distributions in soil. Proc. R. Soc. A Math. Phys..

[33] Hentschel H.G.E., Procaccia I. (1983). The infinite number of generalized dimensions of fractals and strange attractors. Phys. D.

[34] Chhabra A., Jensen R.V. (1989). Direct determination of the *f*(α) singularity spectrum. Phys. Rev. Lett..

[35] Deng L., Liu G., Shangguan Z. (2014). Land-use conversion and changing soil carbon stocks in China’s “Grain-for-Green” program: A synthesis. Glob. Chang. Biol..

[36] Fu B.J., Wang Y.F., Lu Y.H., He C.S., Chen L.D., Song C.J. (2009). The effects of land-use combinations on soil erosion: A case study in the Loess Plateau of China. Prog. Phys. Geogr..

[37] Zhang C., Liu G., Xue S., Sun C. (2013). Soil organic carbon and total nitrogen storage as affected by land-use in a small watershed of the Loess Plateau, China. Eur. J. Soil Biol..

[38] Deng L., Shangguan Z., Sweeney S. (2014). “Grain for Green” driven land-use change and carbon sequestration on the Loess Plateau, China. Sci. Rep.-UK.

[39] Liu X., Zhang G., Heathman G.C., Wang Y., Huang C. (2009). Fractal features of soil particle-size distribution as affected by plant communities in the forested region of Mountain Yimeng, China. Geoderma.

[40] Lyu X., Yu J., Zhou M., Ma B., Wang G., Zhan C., Han G., Guan B., Wu H., Li Y. (2015). Changes of soil particle size distribution in Tidal Flats in the Yellow River Delta. PLoS ONE.

[41] Majumder B., Ruehlmann J., Kuzyakov Y. (2010). Effects of aggregation processes on distribution of aggregate size fractions and organic C content of a long-term fertilized soil. Eur. J. Soil Biol..

[42] Zhuang J., McCarthy J.F., Perfect E., Mayer L.M., Jastrow J.D. (2008). Soil water hysteresis in water-stable microaggregates as affected by organic matter. Soil Sci. Soc. Am. J..

[43] Segal E., Shouse P.J., Bradford S.A., Skaggs T.H., Corwin D.L. (2009). Measuring particle size distribution using laser diffraction: Implications for predicting soil hydraulic properties. Soil Sci..

[44] Martín M.A., García-Gutiérrez C., Reyes M. (2009). Modeling multifractal features of soil particle size distributions with Kolmogorov Fragmentation Algorithms. Vadose Zone J..

[45] Trimble S.W., Crosson P. (2000). Land-use—U.S. soil erosion rates—Myth and reality. Science.

[46] Xu G., Li Z., Li P. (2013). Fractal features of soil particle-size distribution and total soil nitrogen distribution in a typical watershed in the source area of the middle Dan River, China. Catena.

[47] Fu B.J., Ma K.M., Zhou H.F., Chen L.D. (1999). The effect of land-use structure on the distribution of soil nutrients in the hilly area of the Loess Plateau, China. Chin. Sci. Bull..

[48] Xia D., Deng Y., Wang S., Ding S., Cai C. (2015). Fractal features of soil particle-size distribution of different weathering profiles of the collapsing gullies in the hilly granitic region, South China. Nat. Hazards.

[49] Dumanski J., Pieri C. (2000). Land quality indicators: Research plan. Agric. Ecosyst. Environ..

[50] Sun W., Shao Q., Liu J. (2013). Soil erosion and its response to the changes of precipitation and vegetation cover on the Loess Plateau. J. Geogr. Sci..

